# Genome-wide segregation of single nucleotide and structural variants into single cancer cells

**DOI:** 10.1186/s12864-017-4286-1

**Published:** 2017-11-25

**Authors:** John Easton, Veronica Gonzalez-Pena, Donald Yergeau, Xiaotu Ma, Charles Gawad

**Affiliations:** 1 Department of Computational Biology, St. Jude Children’s Research Hospital, Memphis, TN 38105 USA; 2 Department of Oncology, St. Jude Children’s Research Hospital, Memphis, TN 38105 USA; 3 Buffalo Institute for Genomics and Data Analytics, University at Buffalo, Buffalo, NY 14260 USA; 4 Present address: MS 1260, Room IA6042, St. Jude Children’s Research Hospital, 262 Danny Thomas Place, Memphis, TN 38105 USA; 5 Present address: Buffalo Institute for Genomics and Data Analytics CBLS, 701 Ellicott St, Buffalo, NY 14203 USA

**Keywords:** Single-cell genomics, cancer evolution, acute lymphoblastic leukemia

## Abstract

**Background:**

Single-cell genome sequencing provides high-resolution details of the clonal genomic modifications that occur during cancer initiation, progression, and ongoing evolution as patients undergo treatment. One limitation of current single-cell sequencing strategies is a suboptimal capacity to detect all classes of single-nucleotide and structural variants in the same cells.

**Results:**

Here we present a new approach for determining comprehensive variant profiles of single cells using a microfluidic amplicon-based strategy to detect structural variant breakpoint sequences instead of using relative read depth to infer copy number changes. This method can reconstruct the clonal architecture and mutational history of a malignancy using all classes and sizes of somatic variants, providing more complete details of the temporal changes in mutational classes and processes that led to the development of a malignant neoplasm. Using this approach, we interrogated cells from a patient with leukemia, determining that processes producing structural variation preceded single nucleotide changes in the development of that malignancy.

**Conclusions:**

All classes and sizes of genomic variants can be efficiently detected in single cancer cells using our new method, enabling the ordering of distinct classes of mutations during tumor evolution.

**Electronic supplementary material:**

The online version of this article (10.1186/s12864-017-4286-1) contains supplementary material, which is available to authorized users.

## Background

Recent technological advancements have enabled the sequencing of genomes of single cells, a technologically challenging process that starts with a single molecule of DNA [1]. By bringing genomics to the cellular level, we have begun to segregate mutations to distinct cellular populations, enabling us to define the population genetic diversity and clonal structures of complex tissues. Initial studies have provided unexpected insights into the contributions of somatic mosaicism to human development and disease [2, 3]. This has been especially true in cancer where intratumor genetic heterogeneity has provided the opportunity to trace back the mutations and mutational processes that resulted in the formation of a malignancy [4, 5].

Contemporary strategies for amplifying and interrogating the genomes of single cells have resulted in the ability to segregate single nucleotide or copy number variants (CNV) into single cells. However, due to the tradeoffs in genome coverage and uniformity using current amplification strategies, we have had limited success comprehensively detecting both variant classes in the same cells [6]. In addition, most strategies for detecting CNV rely on differences in read depth at specific locations relative to a reference, which are only able to detect large regions of alteration [7]. Further, read depth does not provide information on other classes of structural variation (SV), including: translocations, inversions, insertions of novel sequence, or interspersed copy number gains. Isothermal methods that provide sufficient breadth of genomic coverage to identify most single nucleotide variants (SNV) but much less uniformity in coverage depth have a very limited capacity to detect CNV [5]. Quantitative PCR-based methods have been developed to detect both CNV and SNV in the same cell, but they are only able to interrogate a small number of variants due to limitations in the multiplexing of fluorophores, which hampers the investigator’s ability to accurately determine the clonal structures [8, 9].

**Fig. 1:**
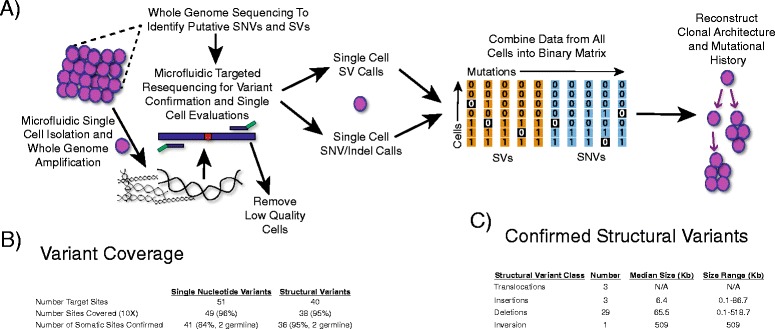
Overview of Experimental Protocol and Performance. **a** Whole genome sequencing is first performed to determine the comprehensive mutation profile of the sample, followed by variant confirmation using targeted resequencing. Single cells are then isolated, followed by amplification of the variant sites, variant calling, and binary matrix construction to determine the clonal structure. **b** Overview of putative variant site coverage and confirmation rates. **c** Class and size of confirmed structural variants

In the present study, we report a strategy for segregating hundreds of any variant type to individual cells in an accurate, cost-effective, and efficient manner. We first perform whole genome sequencing on the bulk sample to comprehensively characterize all types of somatic variation in a bulk population (Fig. 1a). We then amplify the genomes of the single cells using multiple displacement amplification for maximal genome recovery from each cell [10]. This is followed by the detection of SNVs and all classes of SV detected in the bulk sample using amplicon-based resequencing (Fig. 1a). For the SV, we use the breakpoint sequence at the site of rearrangements rather than measuring read depth [11]. To maximize efficiency while reducing reagents costs, we generate the amplicons for single-cell variant calling in the microfluidic devices controlled by the Access Array System, executing thousands of parallel reactions in extremely small volumes in an automated manner. Finally, we use the single cell mutation profile to determine the relationships between cells and infer the clonal structure and mutational history of that malignancy [5, 12].

## Results

As an example to show the capability of this approach to identify all types of variants in the same cells, we segregated mutations into single cells from a sample taken from a child that was diagnosed with acute lymphoblastic leukemia. We first performed whole genome, exome, and RNA sequencing to characterize the somatic variants in that sample as part of the Pediatric Cancer Genome Project [13, 14]. In that patient, we identified 51 putative somatic SNVs and 40 SVs (Fig. 1b, Additional file 1: Table S1, Additional file 2: Table S2). We then performed microfluidic amiplicon-based resequencing to efficiently confirm the bulk mutations using the Access Array Microfluidic System. To minimize effort and cost, generation of amplicons for the confirmation of mutations in the bulk sample was done in the same microfluidic chip with the single cells, with conditions described in detail below.

Using this approach, we were able to cover 96% of the 91 target sites at 10X coverage depth in the bulk samples. We confirmed 36 somatic SVs that included 3 translocations and an inversion, as well as 3 insertions and 29 deletions that ranged in size from 0.1 to 518.7 Kb (Fig. 1c). In addition, we confirmed 41 SNVs that had a strong enrichment for C to T transitions and C to G transversions within an apolipoprotein B mRNA editing catalytic polypeptide-like (APOBEC) motif, in agreement with previous studies of this subtype of leukemia [5, 9].

We then captured and performed whole genome amplification (WGA) of 168 cells in two Fluidigm C1 microfluidic chips where 128 were confirmed to be single cells by microscopy (Additional file 3: Table S3). The genome amplification consistently resulted in the generation of 120-150 ng of product that was harvested in 13 μl of DNA dilution buffer. We then used 3.75 μl of WGA product to perform amplicon-based resequencing of the amplified single cell genomes. To accomplish this, we designed primers to target all putative SNV sites identified in the bulk sequencing using BatchPrimer3 (https://probes.pw.usda.gov/batchprimer3). We used default parameters aiming for amplicons of 100-200 bp that were centered on the putative SNV sites. For SV detection, the assembled breakpoint sequences that were output from CREST [11] were input into batchprimer3. The same primer design criteria were used for SNVs and SVs. If a target site did not have a suitable primer pair, we extended the amplicon length to 300 bp. As detailed in the Access Array manual, we added common sequences ACACTGACGACATGGTTCTACA and TACGGTAGCAGAGACTTGGTCT to the 5′ end of the forward and reverse primers, respectively. Instructions detailed in the C1 for DNA sequencing and Access Array manuals (https://www.fluidigm.com/support) were followed for chip loading, PCR reagents used, and thermocycling conditions. Sequencing was performed on a MiSeq using paired end 150 bp reads.

The reads were then quality trimmed and aligned to hg19 using BWA, which was followed by sorting, duplicate marking, local realignment, and base score recalibration using Picard and GATK. For SNVs, SAMtools was used to create an mpileup, which was followed by variant calling using Varscan. Custom bash scripts then required concordance of the location and base change between the whole genome and confirmation data, as well as a minimum of 3 reads comprising more than 1% of all reads at that position to support the variant call in the single cell. For SV calling, custom bash scripts were used to identify and quantify SV breakpoints in the raw reads. To call an SV breakpoint in a given cell, greater than 40 reads had to be an exact match of the 30 bp that spanned the breakpoint.

**Fig. 2:**
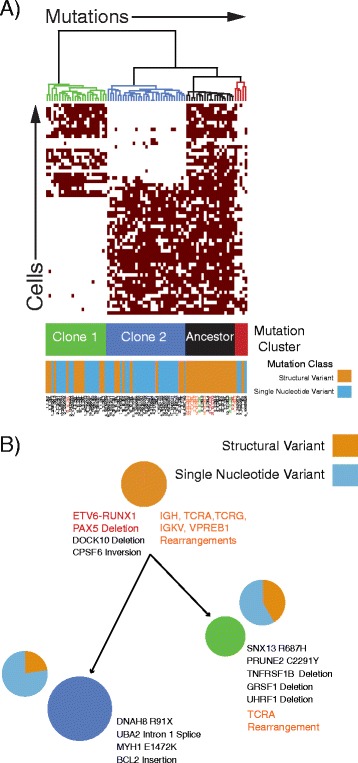
Childhood Leukemia Example. **a** Heatmap depicting clustering patterns of cells and mutations. Three clear mutation clusters segregate into two distinct clonal populations. The ancestral shared mutation cluster is composed entirely of structural variants. **b** Minimal spanning tree showing the relative size of and genetic distance between each clone. Known driver mutations are depicted in red, immune receptor rearrangements in orange, and putative drivers of clonal expansions in black. The relative contribution of single nucleotide and structural variation to each clone are also represented by the pie charts adjacent to each clone

As shown in Fig. 2, after creating a binary matrix of all cells and mutation calls, we then retraced the mutational history of the tumor by grouping cells and mutations into clusters using a mixture model of multivariate Bernoulli distributions. We used our previously published approach, with the addition of SVs in the same binary matrix (https://github.com/lianchye/Clonal_Analysis). We also considered SNVs and SVs to be equivalent contributors to the clonal evolution. This revealed two distinct clonal populations that were formed from 3 distinct mutational clusters. There was one cluster of poor performing assays (red cluster, 6.3% of assays), as well as a small cluster of double cells despite putative identification of single cells by microscopy (red cluster, 9.6% of cells). However, this is likely an overestimate of the number of double cells, as chambers where the amplification started with two genome copies are more likely to produce high quality data that passed our quality control filtering. Closer examination of the mutations in the clusters showed a shared ancestral cluster that had only acquired SV, followed by separate mutation clusters of SNVs and a smaller number of SVs being acquired as the two clones evolved (Fig. 2).

## Discussion

In this study, we present a new method for detecting hundreds of genomic variants in hundreds of single cells in a cost-effective and efficient manner. With this approach, we were able to produce data that further support the assertion that in ETV6-RUNX1 acute lymphoblastic leukemia, there was a distinct process creating SV that drove tumor initiation and preceded the process that induced the later SNVs [15]. In addition, it has been shown that SV in this type of leukemia have a signature of immunoglobulin recombination activating genes (RAG1/2)-mediated rearrangements [16] while the SNVs have an APOBEC signature [15]. Consistent with this, the ancestral clone that only harbored SV had rearrangements of most of the RAG target immune receptor genes. However, that variation was insufficient to produce malignant transformation, which required the APOBEC-mediated SNVs that drove later evolution of the leukemic cell genomes. Thus, with our new method, we are not only able to order the sequences of genetic events, but also the underlying mutational processes that drove malignant transformation of the disease as those normal fetal hematopoietic precursors evolved into leukemia over several years [17]. One limitation of this study is that we rely on bulk sequencing to detect variants. With this approach, we can only detect SNVs and SVs that were present in enough cells to be sampled with bulk sequencing. This allows us to infer the trunk of the clonal structure, but does not provide insights into the intra-clonal evolution and mutational diversity. Further methods developments are needed for the de novo detection of variants in single cells; ideally with high throughput single-cell whole genome or exome sequencing that is executed in an efficient, accurate, and cost-effective manner.

## Conclusions

Single-cell sequencing is a powerful tool for deconvoluting the mutational histories of tumors. Both SNVs and SVs can contribute to malignant transformation, and they frequently occur as a result of distinct mutational processes. By acquiring information on all subtypes and sizes of both variant types in the same cells, we have provided a strategy for determining the order in which those events occurred, and potentially, how they cooperate in the development of human cancer. This strategy can be applied to deconvolute the mutational histories of all types of neoplastic cells as we try to understand the dynamic processes that drive the transformation of normal cells into cancer.

## Methods

### Samples and bulk sequencing

Samples from this patient were obtained as part of the ongoing St. Jude Children’s Research Hospital tissue banking protocol that has been approved by the Institutional Review Board after acquiring written informed consent of the parent if the child was under 16 or the adolescent if they were 16 years or older in accordance with the Declaration of Helsinki. Investigators are blinded to the identity of the participant in this study. Mononuclear cells were isolated from fresh bone marrow samples using Ficoll-Paque (GE Life Sciences), followed by standard cryopreservation. Whole genome, exome, and RNA-sequencing were performed on DNA isolated from the leukemia cells as part of Pediatric Cancer Genome Project [13]. The methods for producing and analyzing the data to generate the putative variant lists have been previously published [14]. We attempted to validate all SVs, as well as SNVs that resided in coding regions.

### Single-cell isolation and WGA

Vials of leukemia cells were thawed using the ThawSTAR System (Biocision), followed by one 15 ml wash in prewarmed RPMI with 1% bovine serum albumin (Sigma). The cells were washed four additional times using Fluidigm wash buffer according to the manufacturer. Cells were filtered using a 15uM strainer (PluriSelect), followed by counting and viability estimation using Luna-FL counter (Logos Biosystems). The cells were then resuspended at a final concentration of 300 cells/ul before mixing with suspension reagent at a ratio of 4ul of cells to 6ul of suspension reagent. The cells were then loaded into a small Fluidigm C1 DNA sequencing microfluidic chip, followed by WGA according to the manufacturer’s instructions.

### Targeted sequencing of variants in single cells

Single cell WGA products underwent targeted sequencing using the Access Array System according to the manufacturer’s instructions as previously reported (Fluidigm) [5]. Primers were designed across SNV sites or SV breakpoints using BatchPrimer3 [18] adding common sequences according to the Access Array instructions (Fluidigm). The sequences of the primers are listed in Additional file 4: Table S4). Bulk DNA from both tumor and a remission sample as a germline control were run on each Access Array chip to confirm bulk variants, as well as to insure successful amplification on the chip. Barcoded amplicons from four Access Array chips were pooled and run on a MiSeq using 2X150bp reads (Illumina).

### Data processing, variant confirmation, and clonal structure determination

Trimming, alignment, and SNV calling were performed as previously reported [5]. SVs were first confirmed in the bulk sample by determining the number of reads that had an exact match to the 30 bp sequence that spanned the breakpoint. Variants were considered present if greater than 40 reads were an exact match of the 30 bp that spanned the breakpoint. That threshold was chosen to minimize false positive calls due to sample cross contamination or incorrect demultiplexing of sequencing reads. Chambers that were visually confirmed to contain one cell and samples that covered at least 80% of target SNV sites at a depth of 10X or greater were retained for further analyses. SV calls were then made in each single cell, which were combined with SNV calls to create a binary matrix containing all cells. The number of clones were estimated after performing hierarchical clustering. Cells were then assigned to clusters using an expectation-maximization algorithm, followed by minimal spanning tree construction as previously reported [5, 12].

## Additional files

Additional file 1: Table S1. List of Somatic Structural Variants Identified in this Sample. This file provides a list of structural variants confirmed in the bulk patient sample, as well as the location, quality, and breakpoint sequence data output by CREST. (XLSX 52 kb)

Additional file 2: Table S2. List of Somatic Single-Nucleotide Variants Identified in this Sample. This file provides a list of single-nucleotide variants confirmed in the bulk patient sample, as well as the location and number of supporting reads output by VarScan. (XLSX 48 kb)

Additional file 3: Table S3. Cell Capture Metrics. This file provides an overview of the number of cells captured and included in the analyses after surpassing quality control criteria, as well as. (PDF 21 kb)

Additional file 4: Table S4. List of Amplicon Primers used in Resequencing. This file provides a list of the primers used for interrogating the bulk and single cell samples for single-nucleotide and structural variants. (XLSX 57 kb)

## Abbreviations

APOBEC: apolipoprotein B mRNA editing enzyme catalytic polypeptide-like
CNV: copy number variation
SNV: single-nucleotide variant
SV: structural variant
WGA: whole genome amplification
RAG: recombination-activating gene.
