# Supplementary material for: Genome-wide segregation of single nucleotide and structural variants into single cancer cells
Source: BMC Genomics. 2017 Nov 25;18:906. doi: 10.1186/s12864-017-4286-1 (PMC5702214; doi:10.1186/s12864-017-4286-1)
Supplement: Supplementary file 3 — Cell Capture Metrics. This file provides an overview of the number of cells captured and included in the analyses after surpassing quality control criteria, as well as. (PDF 21 kb) [file 12864_2017_4286_MOESM3_ESM.pdf]

	Number Single Cells
Captured	168
Visually Confirmed Single Cells	128
Cells with 80% of Sites Covered (10X)	62*

*Estimated False Negative Rate of Included Cells 23.8%
